# Characterization of antennal chemosensilla and associated odorant binding as well as chemosensory proteins in the parasitoid wasp *Microplitis mediator* (Hymenoptera: Braconidae)

**DOI:** 10.1038/s41598-018-25996-3

**Published:** 2018-05-16

**Authors:** Shan-Ning Wang, Shuang Shan, Jing-Tao Liu, Rui-Jun Li, Zi-Yun Lu, Khalid Hussain Dhiloo, Adel Khashaveh, Yong-Jun Zhang

**Affiliations:** 1 Institute of Plant and Environment Protection, Beijing Academy of Agriculture and Forestry Sciences, Beijing, 100097 China; 2 State Key Laboratory for Biology of Plant Diseases and Insect Pests, Institute of Plant Protection, Chinese Academy of Agricultural Sciences, Beijing, 100193 China; 3 College of Plant Protection, China Agricultural University, Beijing, 100193 China; 4 College of Plant Protection, Agricultural University of Hebei, Baoding, 071000 China; 5 IPM Center of Hebei Province, Key Laboratory of Integrated Pest Management on Crops in Northern Region of North China, Ministry of Agriculture, Plant Protection Institute, Hebei Academy of Agricultural and Forestry Sciences, Baoding, Hebei, 071000 China; 6 Department of Entomology, Faculty of Crop Protection, Sindh Agriculture University Tandojam, Hyderabad, 70060 Pakistan

## Abstract

Odorant binding proteins (OBPs) and chemosensory proteins (CSPs) expressed in antennal chemosensilla are believed to be important in insect chemoreception. In the current study, we fully described the morphological characteristics of the antennal sensilla in parasitoid wasp *Microplitis mediator* and analyzed the expression patterns of OBPs and CSPs within the antennae. In *M. mediator*, eight types of sensilla were observed on the antennae. Sensilla basiconica type 2 and s. placodea with wall pores may be involved in olfactory perception, whereas s. basiconica type 1 and type 3 with tip pores may play gustatory functions. Among the 18 OBPs and 3 CSPs in *M. mediator*, 10 OBPs and 2 CSPs were exclusively or primarily expressed in the antennae. *In situ* hybridization experiments indicated that the 12 antennae-enriched OBPs and CSPs were mapped to five morphological classes of antennal sensilla, including s. basiconica (type 1–3), s. placodea and s. coeloconica. Within the antennae, most of OBP and CSP genes were expressed only in one type of sensilla indicating their differentiated roles in detection of special type of chemical molecules. Our data will lay a foundation to further study the physiological roles of OBPs and CSPs in antennae of parasitoid wasps.

## Introduction

Parasitoid wasps, known to locate their suitable hosts in a complex environment are mediated largely by chemical cues. The cues associated with the host can be volatile substances that are perceived by olfaction sensilla at long distances, or contact substances that are mainly perceived by gustatory sensilla at close range^1–4^. Several studies showed that the chemosensory sensilla (olfaction and gustation) used by parasitoid wasps to locate and evaluate their hosts are mostly present on their antennae^4–7^. Olfactory sensilla are characterized by numerous pores on the whole surface, while gustatory sensilla are characterized by terminal or subterminal pores^4,8,9^. The chemical molecules enter into the antennal sensillum cavity through pores, dissolve in the aqueous sensillum lymph, activate the chemosensory receptor neurons, and ultimately leading to behavioral responses^9–12^.

Two families of small soluble proteins, odorant binding proteins (OBPs) and chemosensory proteins (CSPs) are believed to be involved in the chemical communication and perception in insects^13,14^. The both proteins are synthesized in support cells and secreted in the sensillum lymph at extraordinarily high concentrations^15,16^. Although the specific physiological roles of OBPs and CSPs in olfaction and gustation are remained poorly understood, it is widely believed that they play essential roles in transferring semiochemicals across the aqueous sensillum lymph to chemoreceptors in the dendrites of the chemosensory neurons^13,14^. Since the first insect OBPs were reported in the Lepidoptera^16^, a large number of OBP and CSP genes are being identified in different insect species. The copy numbers of OBPs and CSPs in different genomes of insects are highly diversified^17–21^. In each insect species, only a part of OBP and CSP genes have specific or enriched expression in the antennae^17,22,23^, and different OBPs and CSPs are expressed in different antennal sensilla^24–27^.

*Microplitis mediator* (Haliday) (Hymenoptera: Braconidae) is a generalist parasitoid of a wide range of lepidopteran larvae, including *Helicoverpa armigera*, *Mythimna separata* and *Mamestra brassicae*^28,29^. Like most other parasitoid wasps, *M. mediator* uses antennal chemosensilla to detect host-related chemical cues for habitat searching, host location, and host assessment. From the complete antennal transcriptome data of female and male *M. mediator*, in total we identified 20 OBP and 3 CSP genes. As expected, several OBPs and CSPs show higher levels of expression in the antennae^30–32^. Ligand binding characteristics were also investigated among some of these proteins^30,31,33^. So far, the expression of the OBPs and CSPs in the antennae of *M. mediator* has not been systematically assessed. In some parasitoid species, the antennae have been found to contain both olfactory and gustatory sensilla, however, little is known whether OBPs or CSPs are expressed in antennal gustatory sensilla.

In the present study, the antennal morphology and sensilla types of male and female *M. mediator* were characterized. Several types of olfactory and gustatory sensilla were found on the antennae of *M. mediator*. Expression analysis of OBPs and CSPs showed that 10 OBPs and 2 CSPs were expressed mainly in the antennae. Finally, *in situ* hybridization data indicated that the antennal-enriched genes expressed in different types of antennal sensilla of *M. mediator*. Our results will lay a key foundation to further investigate the physiological roles of OBPs and CSPs in parasitic wasp chemoreception.

## Results

### The characterization of *M. mediator* antennae

**Figure 1:**
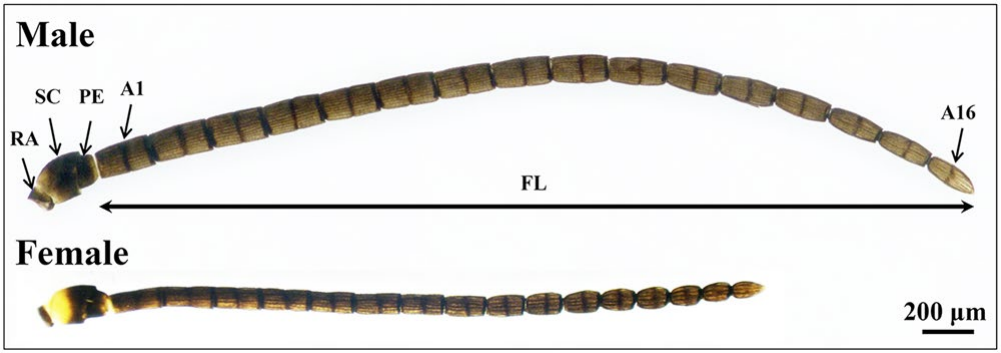
Light microscopic photograph of antennae from *M. mediator*. RA, radicula; SC, scape; PE, pedicel; A1/16, antennomere 1/16; FL, flagellum.

**Figure 2:**
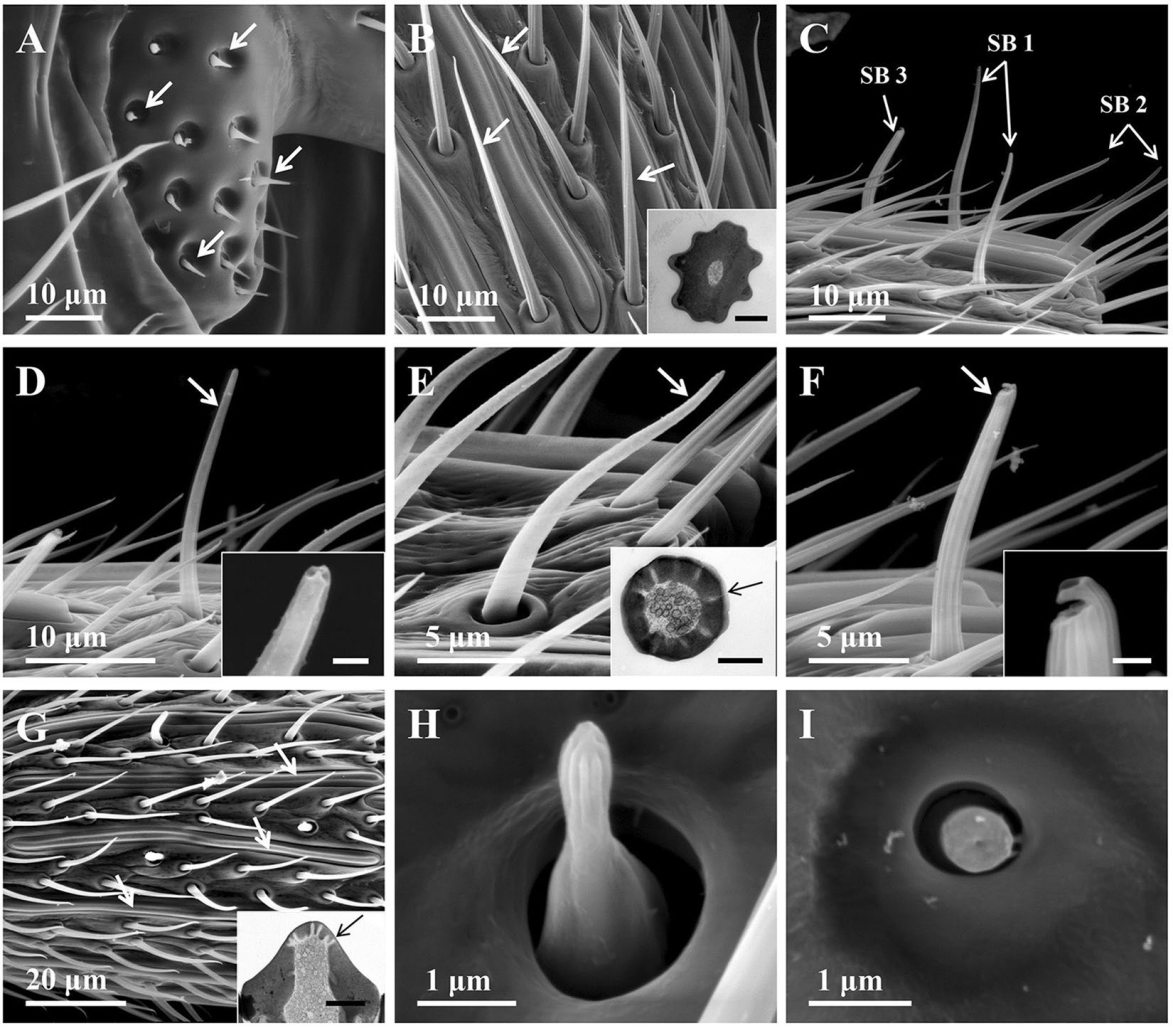
Sensilla identified on the antennae of *M. mediator*. (**A**) S. chaetica on radicula; (**B**) S. trichodea, inset (bar = 0.5 µm): transverse section of a s. trichodea with thick sensillum wall (**C**) Distal part of 14th antennomere of the female antennae with three types of s. basiconica; (**D**) S. basiconica type 1, inset (bar = 0.5 µm): detail of a s. basiconica type 2 with apical pores; (**E**) S. basiconica type 2, inset (bar = 0.5 µm): transverse section of a s. trichodea with multiple pores on wall (arrow); (**F**) S. basiconica type 3, inset (bar = 0.5 µm): detail of the tip of a s. basiconica type 3 with cuticular projection; (**G**) S. placodea, inset (bar = 0.5 µm): transverse section of s. placodea with multiple pores on wall (arrow); (**H**) S. coeloconica; (**I**) S. campaniform. SB1, s. basiconica type 1; SB2, s. basiconica type 2; SB3, s. basiconica type 3.

The antennae of both male and female *M. mediator* are filiform in shape and consist of the basic segments: scape with radicula, pedicel and flagellum (Fig. 1). The flagellum is composed of 16 antennomeres and exhibited strong sexual dimorphism in the length of each antennomere, which are longer in males than in females (Fig. 1). Scanning electron microscopy studies reveal that eight different types of sensilla were identified on the antennae of *M. mediator*, including sensilla chaetica, sensilla trichodea, sensilla basiconica (type 1–3), sensilla placodea, sensilla coeloconica and sensilla campaniform (Fig. 2).

The s. chaetica are found only on the basal portion of the radicula and the pedicel in both males and females. Each sensillum has a smooth cuticle that tapers to a blunt tip, and inserted in the cavities of the antennal socket (Fig. 2A).

The s. trichodea are the most abundant and present in all antennal segments of both sexes. This type of sensillum had a longitudinal grooved surface and sharply pointed tip (Fig. 2B). The sensillar cuticle was thick solid without porous and there was no dendrites of sensory neurons in the sensillum lymph (Fig. 2B).

The s. basiconica occur only on flagellum. Based on their different shapes, they are further divided into three subtypes: s. basiconica type 1, s. basiconica type 2 and s. basiconica type 3 (Fig. 2C). S. basiconica type 1 are distributed more in the middle and distal of antennal segments and more easily visualized at the apex of the last antennomere of both sexes. Each sensilum has a grooved surface and a blunt tip with apical pores (Fig. 2D). S. basiconica type 1 are oriented more perpendicularly with the antennae axis than s. trichodea and s. basiconica type 2 (Fig. 2D). S. basiconica type 2 are the most abundant among s. basiconica. They are found on all flagellar segments of both sexes. This type of sensillum has smooth cuticular surfaces and gradually curved with blunt tip (Fig. 2E). The cuticular wall is penetrated by numerous pores, and surrounded by numerous dendritic branches within the lumen (Fig. 2E). S. basiconica type 3 are only observed in the females, and are easily visualized on the ventral surface of the middle and proximal antennal segments. Similar to s. basiconica type 1, s. basiconica type 3 exhibited a longitudinally grooved and aligned perpendicular to the antennae axis (Fig. 2F). Each sensillum is shorter than s. basiconica type 1, and could be characterized by a sub-terminal pore that opens backwards (Fig. 2F).

The s. placodea are on all flagellar segments of both sexes. They are slightly elevated above the flagellar surface and are distributed into two regular rings along the longitudinal axis of the antennomere (Fig. 2G). This sensillar type had a smooth and porous surface. The sensillum lumen was innervated by numerous dendritic branches (Fig. 2G).

The s. coeloconica are found on the mid dorsal surface of antennomere 4–15 on the male antenna (one or two on each) and 6–15 on the female antenna (only one on each). They are located in depressions and surrounded by a ring-like cuticular elevation (Fig. 2H). These sensilla are peg-like in shape and terminate in a bulb-like tip (Fig. 2H).

The s. campaniform are only observed on the apex of some odd number antennomeres (only one on each) in males. This type of sensilla had an oval donut shape with a pore at the center of the tip (Fig. 2I).

### Expression profiles of *M. mediator* OBP and CSP genes

**Figure 3:**
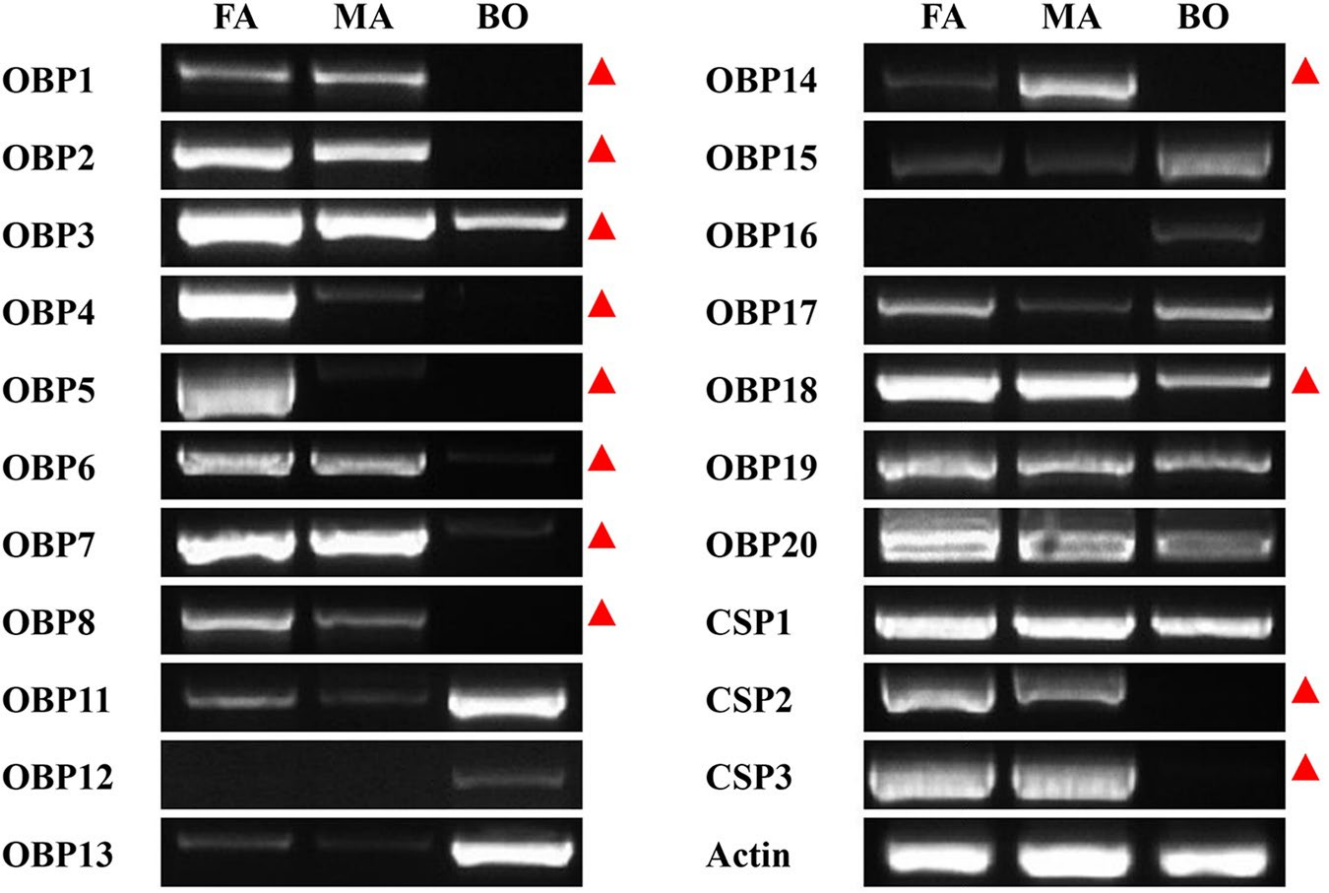
RT-PCR analysis of OBPs and CSPs in different tissues of *M. mediator*. FA: female antennae; MA: male antennae; BO: body. Antennae specific or enriched genes were labeled with a red triangle. *β-actin* was used as a reference gene. The RT-PCR measurements for all genes were performed under the same experimental conditions. The gels of each gene are cropped from different gels. The full-length gels are presented in Supplementary Fig. S1.

Except two almost identical OBPs (OBP9 and OBP10), the expression patterns of the 18 OBPs and 3 CSPs in different tissues (female antennae, male antennae and body) were assessed by semi-quantitative reverse transcription polymerase chain reaction (RT-PCR) (Fig. 3). The actin gene was constitutively expressed in all three tissues, which could provide a stable control for the integrity of the cDNA templates. RT-PCR results indicated that 10 OBP genes (OBP1, OBP2, OBP3, OBP4, OBP5, OBP6, OBP7, OBP8, OBP14 and OBP18) and 2 CSPs (CSP2 and CSP3) were exclusively or primarily expressed in the antennae. Among these genes, OBP4 and OBP5 had female antennae-specific expression whereas OBP14 showed a male antennae-enriched expression. Four genes (OBP17, OBP19, OBP20 and CSP1) were expressed in both the antennae and body part. However, five OBPs, namely OBP11, OBP12, OBP13, OBP15 and OBP16, were expressed mainly in the body. These genes had low expression level (OBP11, OBP13 and OBP15) or no expression (OBP12 and OBP16) in the antennae.

### *In situ* hybridization of OBPs and CSPs within antennae

The precise expression pattern of the 12 antennae-enriched genes (10 OBPs and 2 CSPs) was further determined by *in situ* hybridization. Longitudinal sections and cross sections through the female and male antennae were hybridized with Dig-labelled RNA probes. Using the antisense probes, all of the antennae-enriched OBP and CSP genes were detected in the antennae. Two body-enriched OBPs (OBP11 and OBP13) which were selected as control genes could not be detected in both the female and male antennae using antisense probes (see Supplementary Fig. S1). Expression of the antennae-enriched genes was observed in each of the five morphological classes of sensilla, including s. basiconica type 1, s. basiconica type 2, s. basiconica type 3, s. placodea and s. coeloconica (Table 1).

**Table 1:** Expression characteristics of OBPs and CSPs in sensilla on antennae of *M. mediator*.

Genes	Female antennae					Male antennae			
	SB 1	SB 2	SB 3	SP	SC	SB 1	SB 2	SP	SC
OBP1		√					√		
OBP2				√				√	
OBP3	√		√			√			
OBP4			√						
OBP5			√						
OBP6				√				√	
OBP7				√				√	
OBP8					√				√
OBP14								√	
OBP18	√					√			
CSP2					√				√
CSP3		√					√		

SB1, s. basiconica type 1; SB2, s. basiconica type 2; SB3, s. basiconica type 3; SP, s. placodea, SC, s. coeloconica; √, expressions were detected in the sensilla.

**Figure 4:**
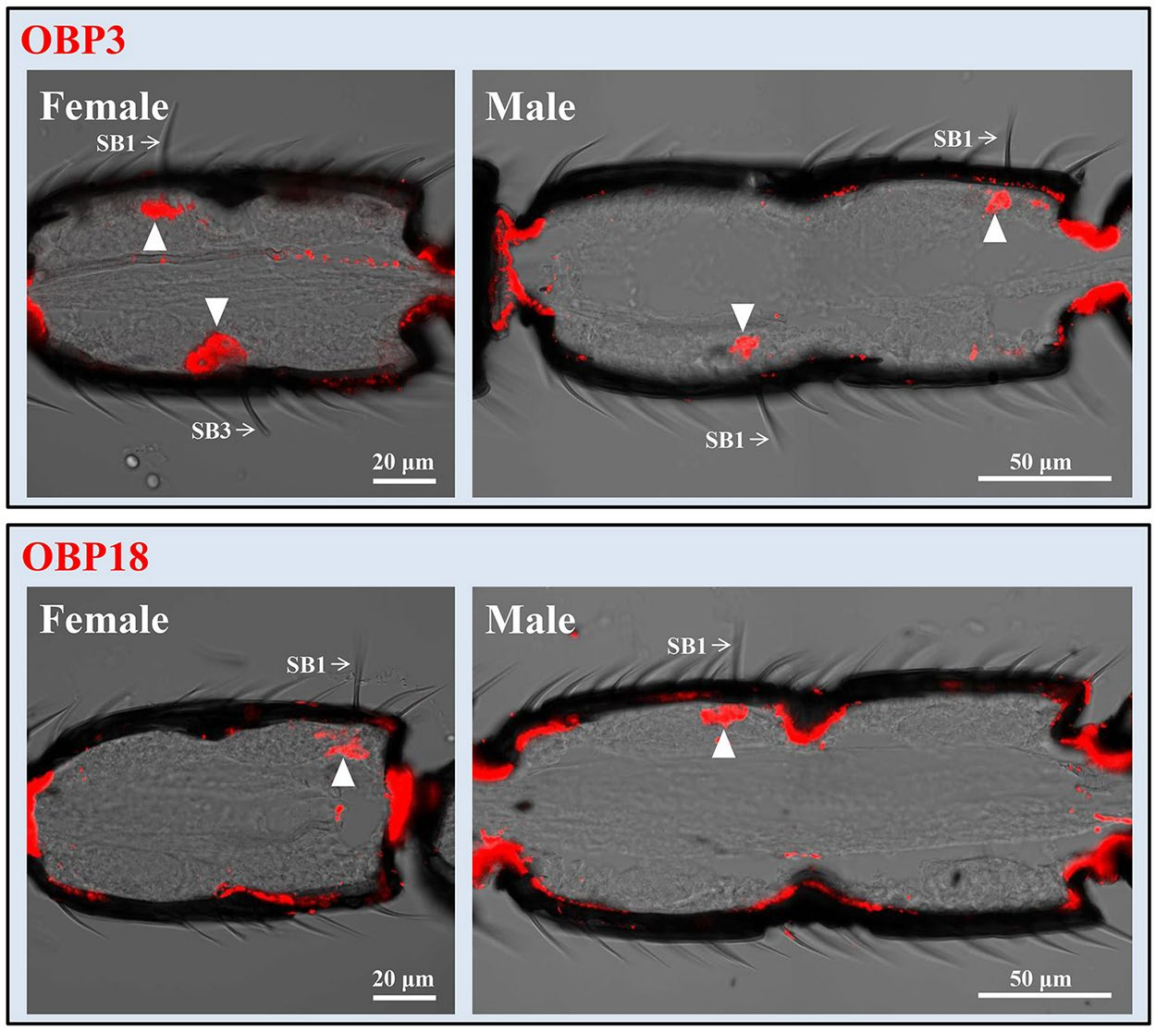
*In situ* hybridization of OBP3 and OBP18 in antennae of *M. mediator*. Dig-labelled antisense RNA probes for OBPs were hybridized to longitudinal sections through the female and male antennae and visualized by red fluorescence. OBP3 probe labeled cells were located at the base of the s. basiconica type 1 and type 3 in female antennae. In male antennae, the labeled cells were located at the base of s. basiconica type 1. OBP18 positive cells were located at the base of the s. basiconica type 1 on female and male antennae.

**Figure 5:**
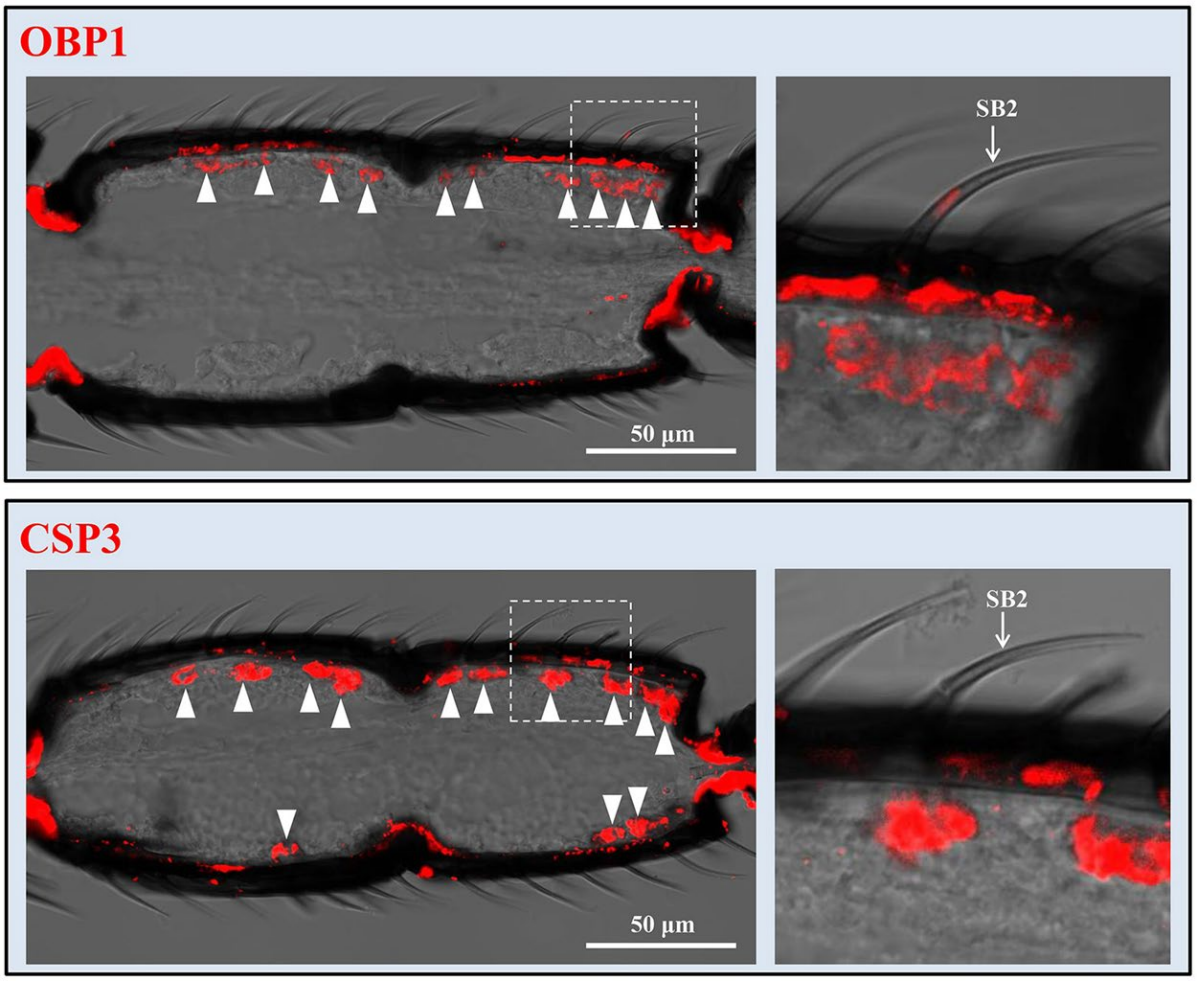
Localization of OBP1 and CSP3 in the s. basiconica type 2 on antennae of *M. mediator*. Longitudinal sections show the distribution of OBP1 and CSP3 probe labeled cells (triangles) in the male antennae. Boxed areas are shown at higher magnification on the right. The labeled cells were detected under the s. basiconica type 2 (arrows).

**Figure 6:**
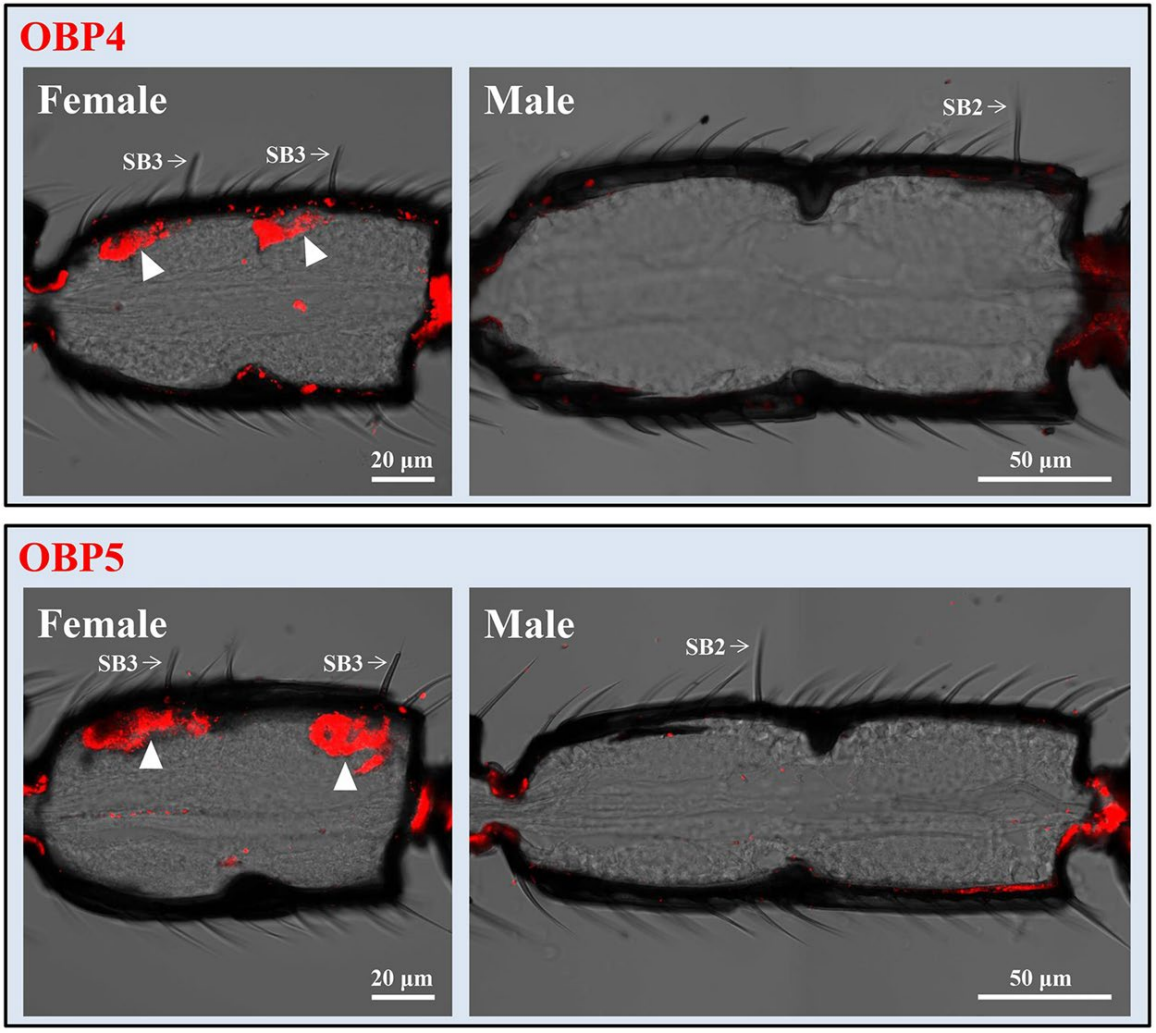
*In* *situ* hybridization assays of OBP4 and OBP5 in antennae of *M. mediator*. Longitudinal sections show the distribution of OBP4 and OBP5 probe labeled cells in the female and male antennae. In female antennae, the labeled cells were located at the base of s. basiconica type 3 (arrows). No hybridization signals were detected in the male antennae. SB2, s. basiconica type 2; SB3, s. basiconica type 3.

OBP3 and OBP18 were expressed in s. basiconica type 1 of both male and female antennae (Fig. 4). Of these two OBPs, OBP3 was also expressed in s. basiconica type 3 on female antennae (Fig. 4). OBP1 and CSP3 were expressed in s. basiconica type 2 (Fig. 5). No sexual dimorphism was observed between male and female antennae except that males appeared to show stronger labelling than females with OBP1 and CSP3 probe under s. basiconica type 2. OBP4 and OBP5 were expressed in s. basiconica type 3 (Fig. 6). Unlike OBP3, OBP4 and OBP5 were not expressed in s. basiconica type 1 on male antennae (Fig. 6).

**Figure 7:**
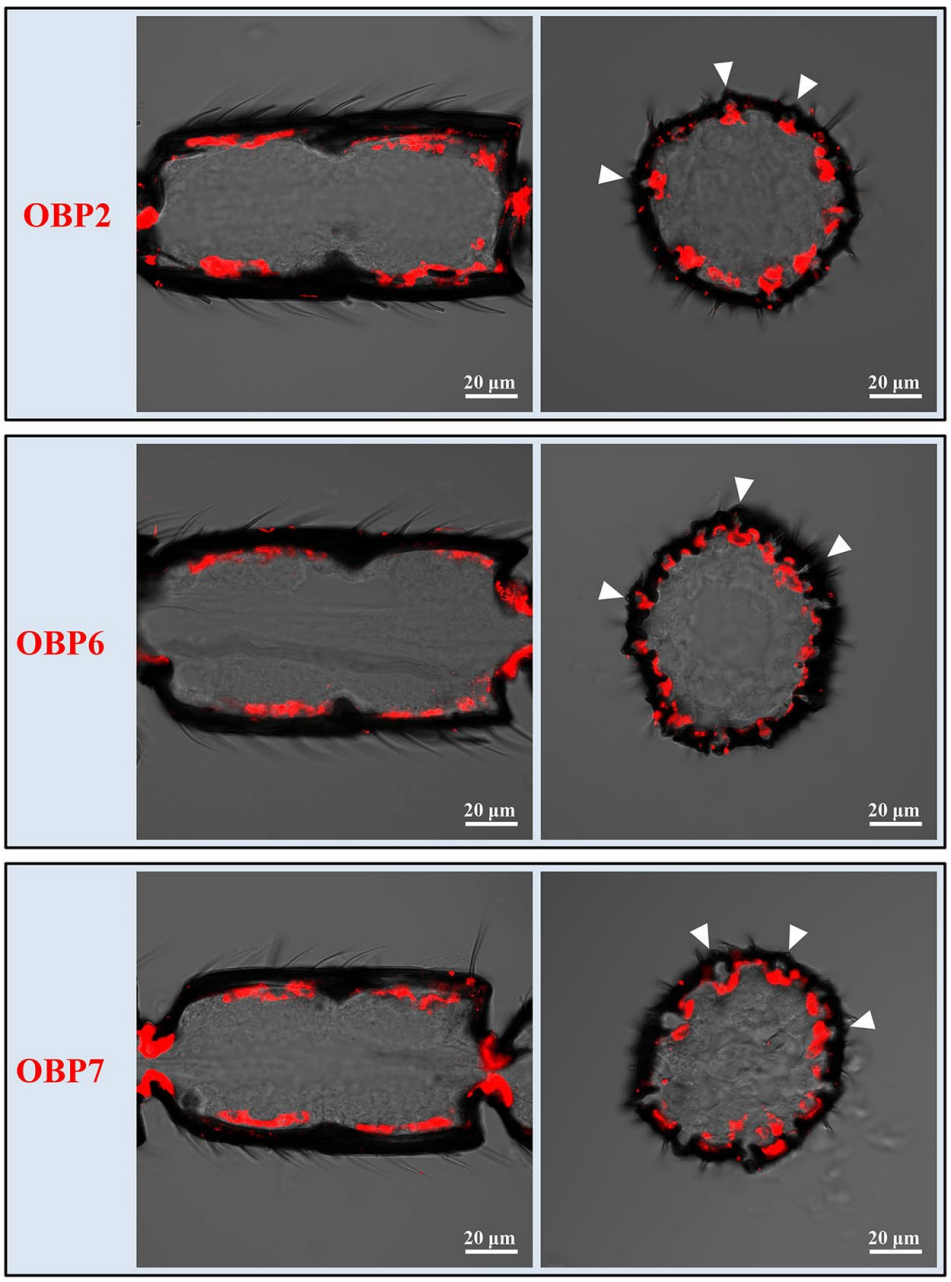
Expression profiles of OBP2, OBP6 and OBP7 in the s. placodea on antennae of *M. mediator*. Longitudinal sections (left panel) show the distribution of OBP2, OBP6, and OBP7 in the female antennae. Cross sections (right panel) show the labeled cells were detected under the s. placodea (triangles).

**Figure 8:**
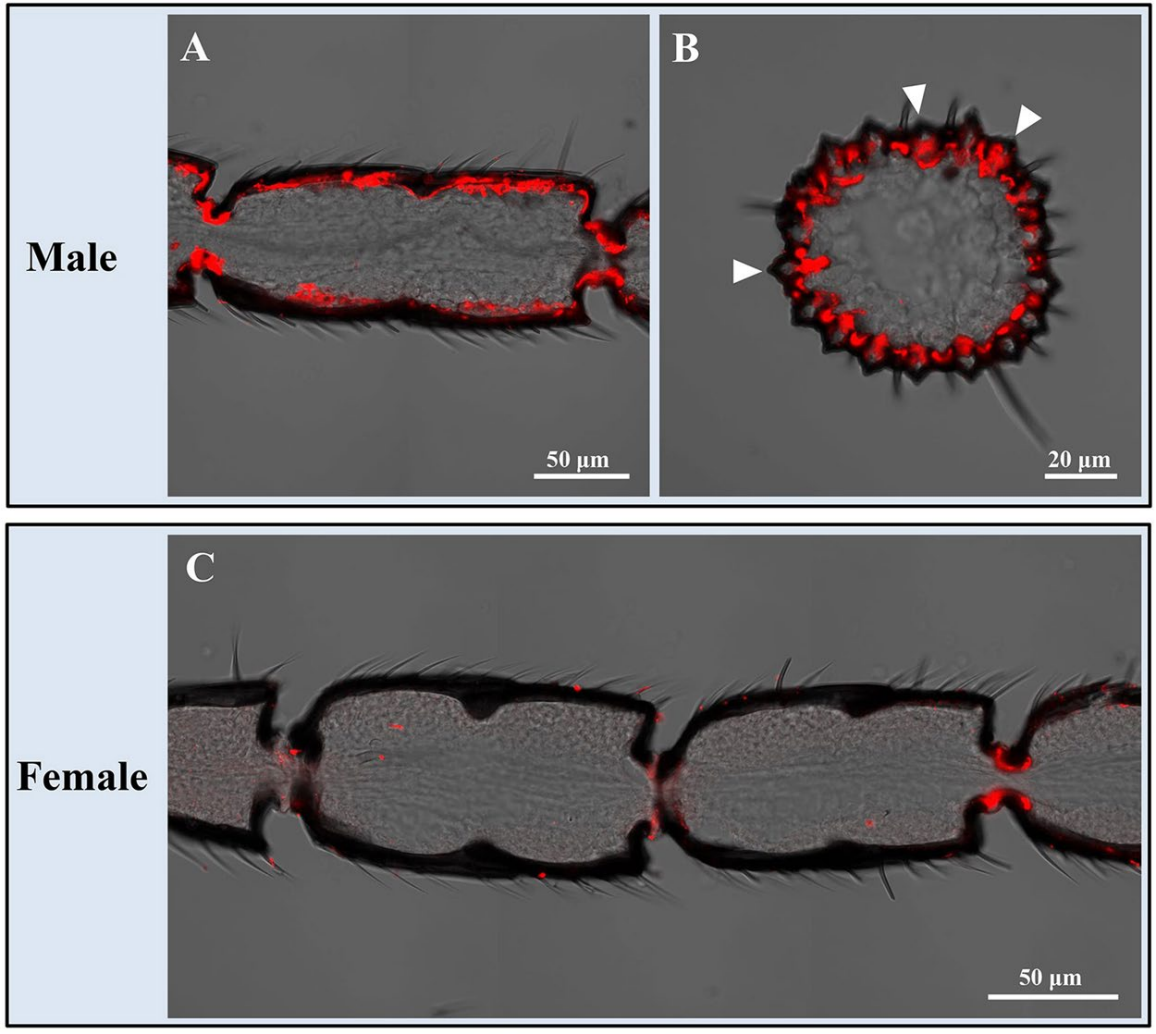
Sex-biased expression of OBP14 in the s. placodea on antennae of *M. mediator*. (**A**) A longitudinal sections show the distribution of OBP14 in the male antennae. (**B**) A cross sections show the OBP14 probes labelled cells under the s. placodea (triangles). (**C**) No hybridization signals were observed in the female antennae.

Four genes including OBP2, OBP6, OBP7 and OBP14 were expressed in s. placodea (Figs 7, 8). In longitudinal sections, the distribution patterns of the hybridization signals for these genes resemble the spatial arrangement of s. placodea along the antenna (Figs 7, 8). In cross sections, labelling could be clearly associated with numerous s. placodea (Figs 7, 8). Of the four genes in s. placodea, OBP14 was only detected in the male antennae (Fig. 8), others were expressed in both the male and female antennae.

**Figure 9:**
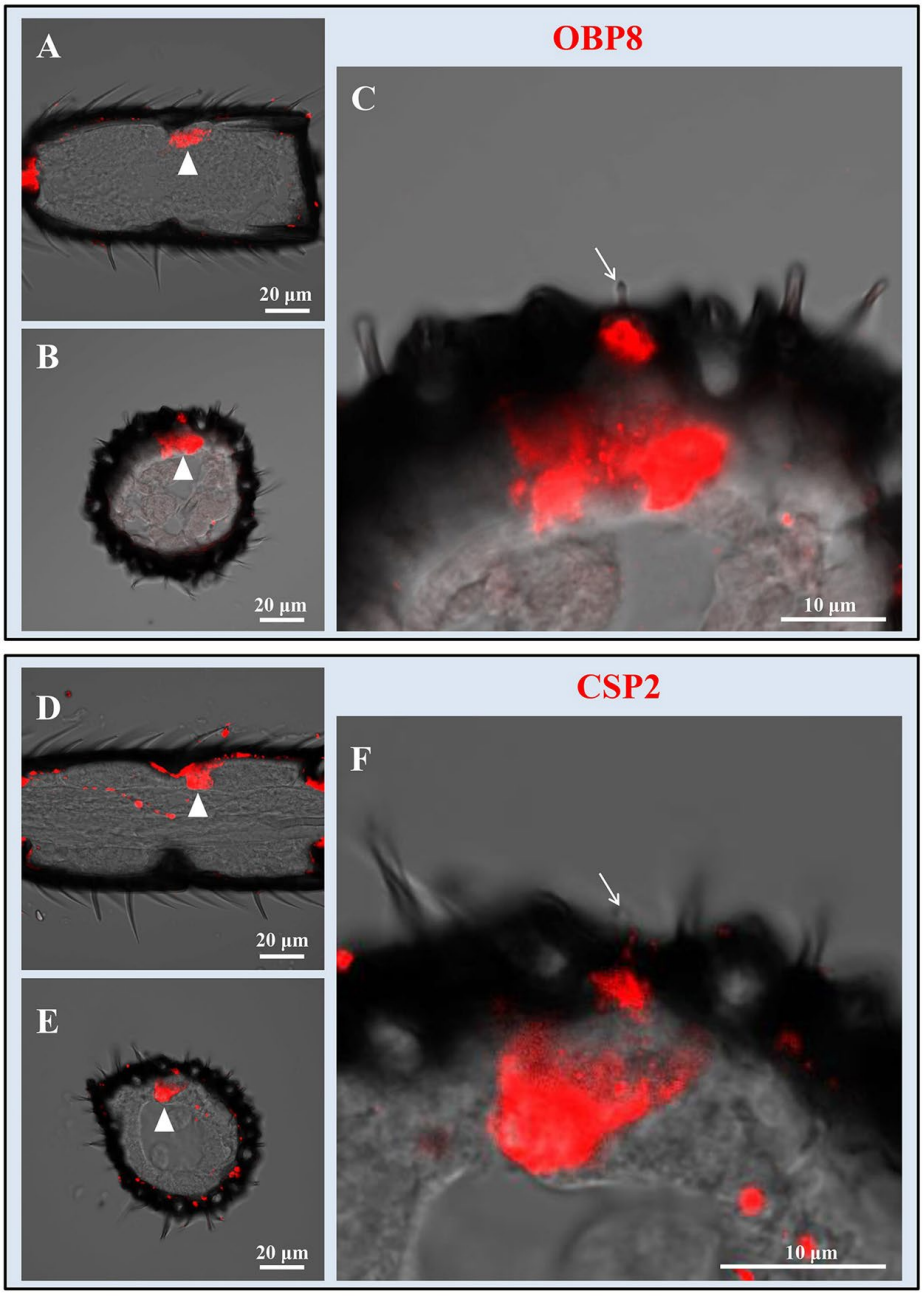
Expression characteristics of OBP8 and CSP2 in the s. coeloconica on antennae of *M. mediator*. Longitudinal sections (**A**,**D**) and cross sections (**B**,**E**) show the distribution of OBP8 and CSP2 in the female antenna. Higher magnifications (**C**,**F**) show the labeled cells of OBP8 and CSP2 under the bases of s. coeloconica (arrows).

**Figure 10:**
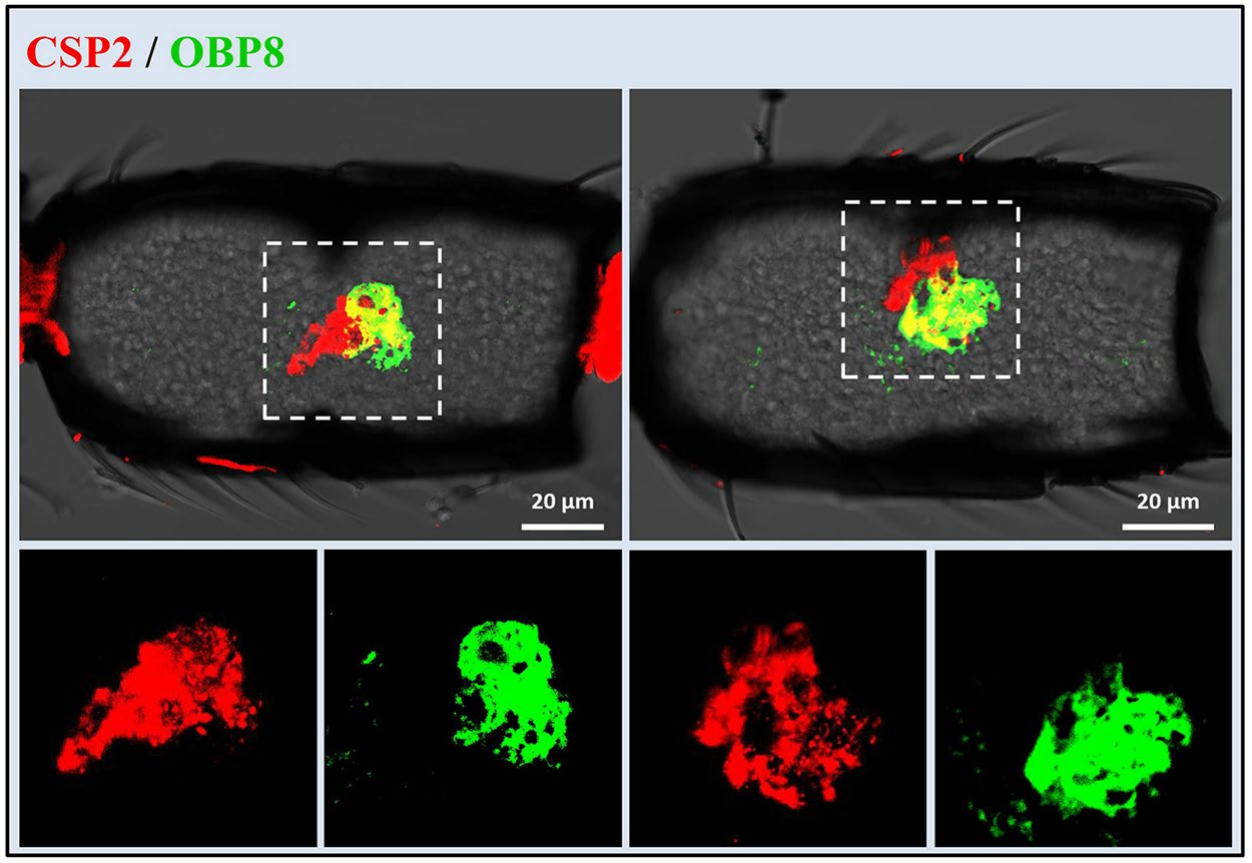
Co-localization of OBP8 and CSP2 in the antennae of *M. mediator*. Two antennal segments of the longitudinal sections show the expression of OBP8 (green) and CSP2 (red) in female antennae of *M. mediator*. In boxed areas (up), the red and green fluorescence channels are shown separately (down).

OBP8 and CSP2 were expressed in s. coeloconica on both male and female antennae. In longitudinal and cross sections, hybridization signals of OBP8 and CSP2 were only observed at the bases of the s. coeloconica (Fig. 9). In two color *in situ* hybridization, OBP8 and CSP2 were expressed in same region with partial overlay, which further was demonstrated that both two genes were expressed in the same sensilla (Fig. 10).

## Discussion

In the present work, eight different types of sensilla were recorded on the antennae of *M. mediator*. Seven of these sensilla types were described in another parasitic wasp, *Microplitis croceipes*^34,35^. Generally, the putative function of sensilla can be deduced from the number of pores and internal dendrites. Non-porous s. trichodea and s. chaetica are considered to be mechanoreceptors^35–38^. S. placodea and s. basiconica type 2 with multiple wall pores and numerous dendrites had been known as olfactory sensilla, while s. basiconica type 1 and type 3 with terminal pores are considered to be gustatory sensilla^35,37,38^. S. coeloconica may play roles in olfaction of wasps, but some researchers also suggested these sensilla were probably associated with thermo-hygro perception^4,35,37,38^. S. campaniform was also described as s. coeloconica type 2, however, the main functions of these sensilla is still unknown^7,36^.

Insect olfactory sensilla play important roles in detection of volatile semiochemicals at a long distance during the initial steps of the host location process. S. placodea were observed on antennae of many parasitic wasp species^4^. In electrophysiological recording assays, Ochieng *et al*.^35^ confirmed that s. placodea were involved in olfactory perception of *M. croceipes*. In the present work, three OBPs (OBP2, OBP6 and OBP7) were expressed in this type of sensilla from both sexes of *M. mediator*, indicating their perception roles of volatiles. Indeed, our previous study proved that the three recombinant OBPs could bind several plant odorants^33^. S. placodea with higher numbers in the male parasitoid wasps may be involved in mate location, especially in the detection of sex-pheromones^4,35,36,38^. Interestingly, OBP14 was only expressed in s. placodea on the male antennae of *M. mediator*, suggesting that OBP14 may play a role in sex-pheromone perception in *M. mediator*. S. basiconica type 2 have been considered as putative olfactory sensilla in many insects^35,37,38^. OBP1 and CSP3 were expressed in the s. basiconica type 2, suggesting their functions in detection of volatiles. The olfactory function of s. coeloconica is still unclear in parasitoid wasps^35^. However, OBP8 and CSP2 were expressed in the s. coeloconica, indicating their putative chemoreception roles in *M. mediator*.

Although OBPs were originally identified in olfactory sensilla^16^, they were also expressed in the gustatory sensilla and involved in gustatory perception^23,39–41^. Our *in situ* data showed that four OBPs (OBP3, 4, 5, and 18) were expressed in gustatory sensilla. In parasitoid wasps, gustatory sensilla play roles in detection of non-volatile contact chemicals. Females use the gustatory sensilla to discriminate between different host species as well as to determine if a host was already parasitized by other wasps^3,4,42^. OBP4 and OBP5 were specifically expressed in female antennae and may play critical roles in detection of the gustatory stimuli which are associated with the recognition and acceptance of the host for the female *M. mediator*.

In conclusion, several types of olfactory and gustatory sensilla on the antennae of *M. mediator* were identified, each of them being probably involved in the detection of different types of chemical cues. OBPs and CSPs of *M. mediator* were expressed in five morphological classes of sensilla and most of them were expressed only in one sensilla type. It seems likely that each of them could evolve to meet the requirement of individual sensilla in the detection of one particular type of chemical. Overall, our data provides basic information for further research regarding to the chemosensory mechanisms involved in host recognition and mating behavior in parasitoid wasps. In future study, we will investigate the detailed roles of the antennal gustatory sensilla by using electrophysiological recordings and identify the ligands binding to gustatory-expressed OBPs of *M. mediator*.

## Methods

### Insects

The cocoons of *M. mediator* were obtained from Plant Protection Institute, Hebei Academy of Agriculture and Forestry Sciences. Adult wasps hatched in a growth chamber maintained at 28 ± 1 °C, 60 ± 10% relative humidity and a 16 L: 8D photoperiod. Newly emerged adults were given 10% sucrose solution.

### Observation of sensilla on the antennae of *M. mediator*

In light microscopic observation, the antennae of both sexes of *M. mediator* were removed and kept in 10% NaOH solution for 30 min in 100 °C, and washed for 5 min in 10% acetic acid. The antennae were then rinsed in ethanol series (70%, 80%, 90%, 95%, 100% ethanol, for 10 min each; 100%, 95%, 90%, 80%, 70% ethanol, for 10 min each) and mounted on a coverslip in Mowiol solution (10% polyvinyl alcohol 4–88, 20% glycerol in PBS). Preparations were analyzed with a digital microscopy (VHX-2000, Keyence, Osaka, Japan).

In scanning electron microscopy observation, the heads of both sexes *M. mediator* were removed from new emergence adults and kept in 70% ethanol for 3 h, and then dehydrated in ethanol series (80%, 85%, 90%, 95%, 100% ethanol, for 10 min each) before undergoing critical point drying. The samples were mounted on a holder using double-sided sticky tapes, sputter coated with gold/palladium and viewed with a scanning electron microscope (Quanta 200 F, FEI, Oregon, USA).

In transmission electron microscopy assay, the antennae of both sexes of *M. mediator* were removed and fixed in a mixture of paraformaldehyde (4%), glutaraldehyde (2%) and sucrose (5%) in 0.1 M PBS (pH = 7.4) for 24 h. After dehydrated in ethanol series, the antennae were embedded in Epon and polymerized in Epon at 60 °C for 48 h. Ultrathin sections (60–80 nm) were cut with a diamond knife, collected on formvar-coated copper grids and stained with uranyl acetate and lead citrate. The samples were observed in a transmission electron microscope (H-7500, Hitachi, Tokyo, Japan).

### RT-PCR analysis

RT-PCR was performed using cDNA prepared from the female antennae, male antennae and body part (mixture of heads, thoraxes, abdomens, legs, wings). RNAs were extracted using the Trizol reagent (Invitrogen, Carlsbad, CA, USA) and corresponding cDNA were synthesized using the Fast Quant RT kit (TIANGEN, Beijing, China) following the manufacturer’s protocol. Primers were designed using the Primer 3 and sequences are listed in Supplementary Table S1. Each PCR reaction (25 μL volume) contained 200 ng of cDNA from different tissues as a template. The cycling conditions were 95 °C for 4 min with 30 cycles as follows: 94 °C for 30 s, 55 °C for 30 s, and 72 °C for 45 s. The final extension step was at 72 °C for 10 min. The amplification products were checked on 1.2% agarose gels. For each gene, one amplification product was cloned into the pEasy-T3 vector (TransGen, Beijing, China) and was sequenced to confirm the identity.

### RNA probe synthesis and *in situ* hybridization

Plasmids containing the coding regions of OBP and CSP genes (see Supplementary Table S2) were linearized with *Sac* I or *Sal* I (TaKaRa, Dalian, China). Digoxigenin (DIG)-labeled and biotin-labeled RNA probes were synthesized using DIG RNA Labeling Kit (SP6/T7) and Biotin RNA Labeling Mix (Roche, Mannheim, Germany) following the protocols.

RNA *in situ* hybridization was performed as previously described^43^. In brief, antennae were dissected and embedded in Tissue-Tek O.C.T. compound (Sakura Finetek, Torrance, USA). Antennal cryosections (12 μm) were collected on Superfrost Plus microscope slides (Fisher Scientific, USA). Sections were fixed at 4 °C and pre-treated at room temperature. After pre-hybridized, the sections were incubated with hybridization solution containing RNA probes at 60 °C overnight. Detection of DIG-labeled probes was performed by using an anti-Dig AP-conjugated antibody (Roche) in combination with HNPP/Fast Red (Roche); a streptavidin-HRP (Perkin Elmer, USA) and Fluorescein Tyramide (TSA, Perkin Elmer, USA) were used for simultaneous detection of biotin-labeled probes. The sections were analyzed using a Zeiss LSM 880 laser scanning microscope (Zeiss, Oberkochen, Germany) and images were processed with ZEN 2012 software.

## Electronic supplementary material

Supplementary Information
