# Supplementary material for: Characterization of antennal chemosensilla and associated odorant binding as well as chemosensory proteins in the parasitoid wasp Microplitis mediator (Hymenoptera: Braconidae)
Source: Sci Rep. 2018 May 16;8:7649. doi: 10.1038/s41598-018-25996-3 (PMC5955942; doi:10.1038/s41598-018-25996-3)
Supplement: Supplementary file 1 — Supplementary Information [file 41598_2018_25996_MOESM1_ESM.pdf]

Supplementary Information for

Characterization of antennal chemosensilla and associated odorant binding as well as chemosensory proteins in the parasitoid wasp *Microplitis mediator* (Hymenoptera: Braconidae)

Shan-Ning Wang[1,2], Shuang Shan[2,3], Jing-Tao Liu[2], Rui-Jun Li[4], Zi-Yun Lu[5], Khalid Hussain Dhiloo[6], Adel Khashaveh[2], Yong-Jun Zhang[2]*

[1] *Institute of Plant and Environment Protection, Beijing Academy of Agriculture and Forestry Sciences, Beijing 100097, China*

[2] *State Key Laboratory for Biology of Plant Diseases and Insect Pests, Institute of Plant Protection, Chinese Academy of Agricultural Sciences, Beijing 100193, China*

[3] *College of Plant Protection, China Agricultural University, Beijing 100193, China*

[4] *College of Plant Protection, Agricultural University of Hebei, Baoding 071000, China*

[5] *IPM Center of Hebei Province, Key Laboratory of Integrated Pest Management on Crops in Northern Region of North China, Ministry of Agriculture, Plant Protection Institute, Hebei Academy of Agricultural and Forestry Sciences, Baoding, Hebei 071000, China*

[6] *Department of Entomology, Faculty of Crop Protection, Sindh Agriculture University Tandojam, 70060，Pakistan*

*Corresponding author

Yong-Jun Zhang, PhD. State Key Laboratory for Biology of Plant Diseases and Insect Pests, Institute of Plant Protection, Chinese Academy of Agricultural Sciences, Beijing, 100193, China. Email: yjzhang@ippcaas.cn; Tel.:+86 10 62815929; Fax: +86 10 62816631.

Supplementary Figures

Figure S1. The full-length gels for each genes presented in Figure 3.

Figure S2. Expression profiles of OBP11 and OBP13 in female and male antennae of *M. mediator*. No hybridization signal was detected in the antennae hybridized with Dig-labelled antisense RNA probes for OBP11 and OBP13.

Supplementary Tables

Table S1. Primers used in RT-PCR analysis.

Table S2. OBP sequences of *M. mediator* used as probes in *in situ* hybridization.

Figure S1.

OBP1

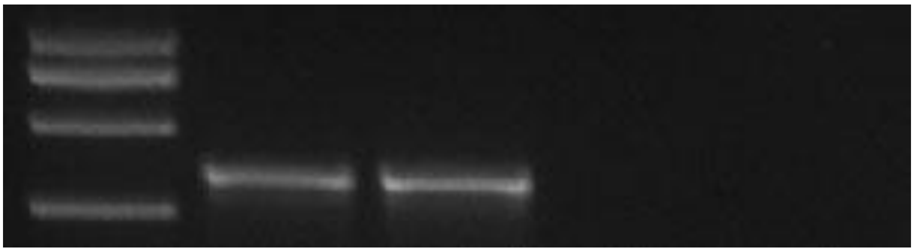

OBP2

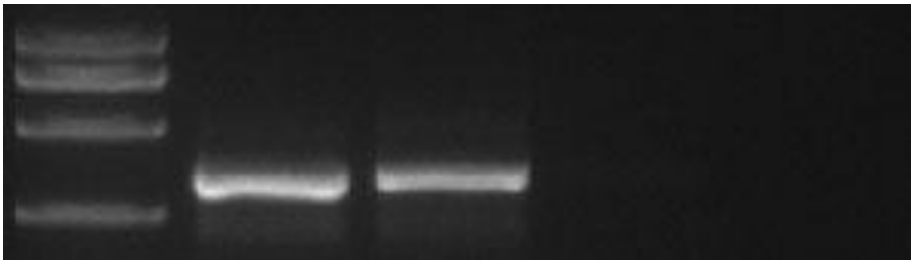

OBP3

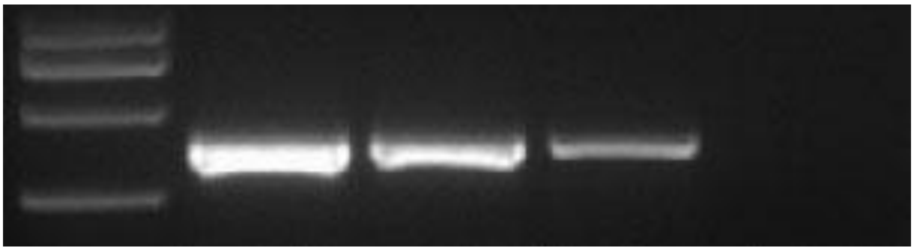

OBP4

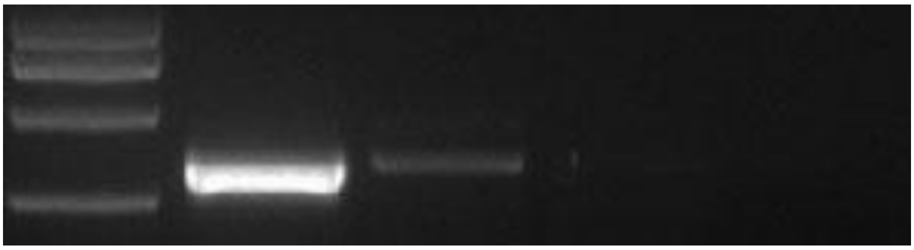

OBP5

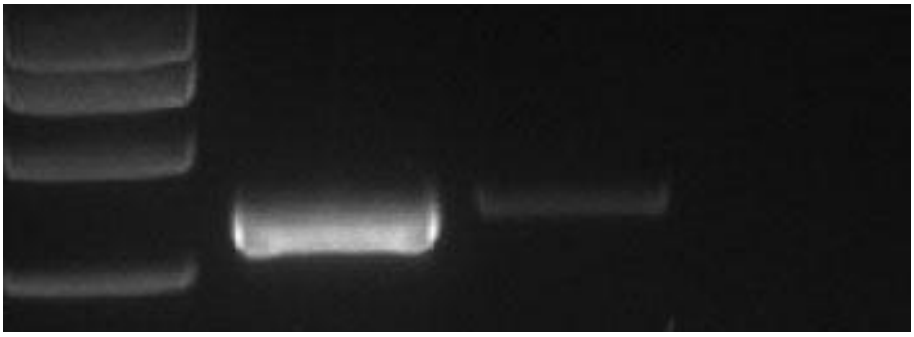

OBP6

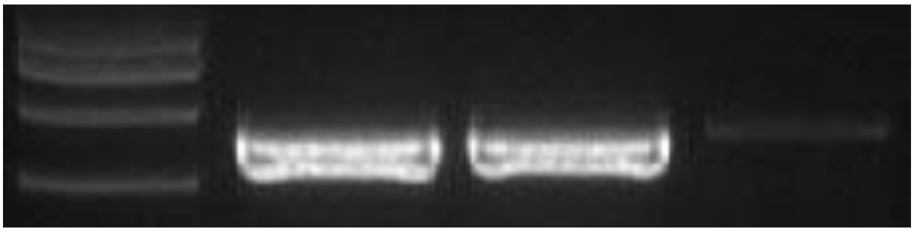

OBP7

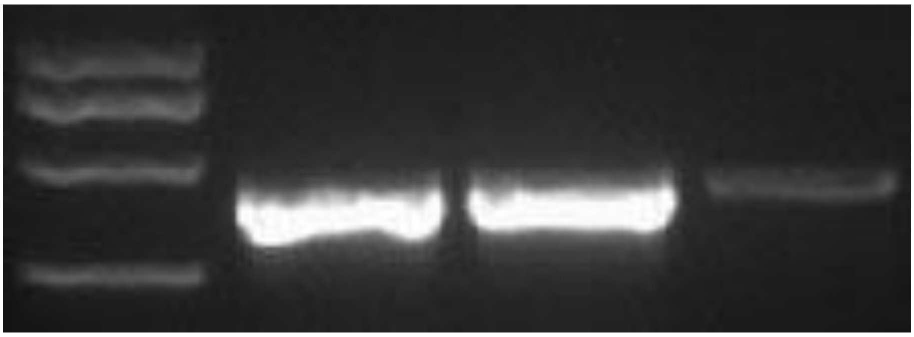

OBP8

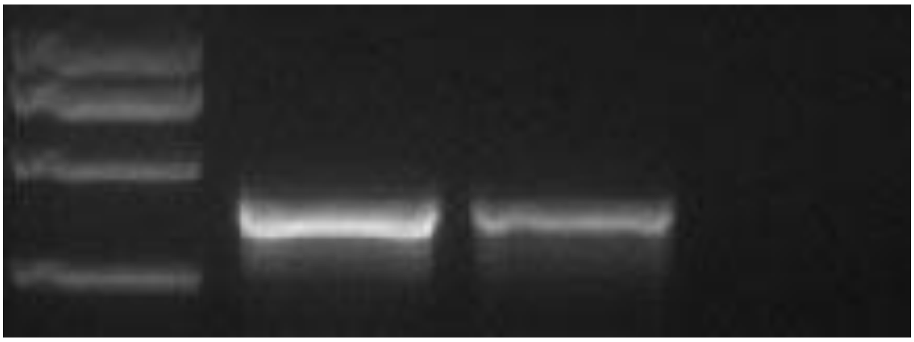

OBP11

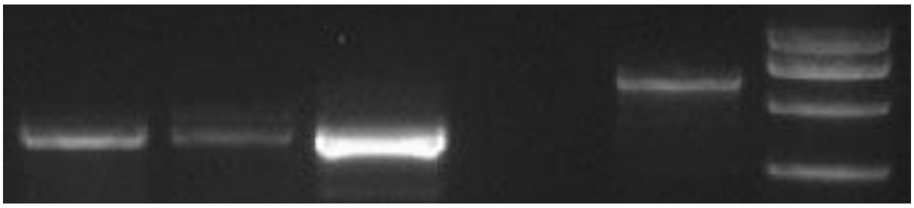

OBP12

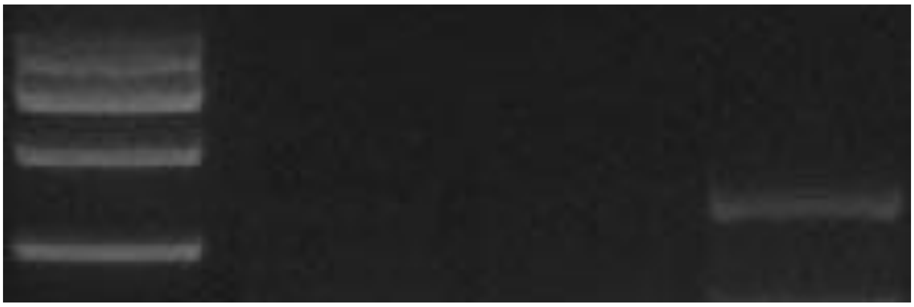

OBP13

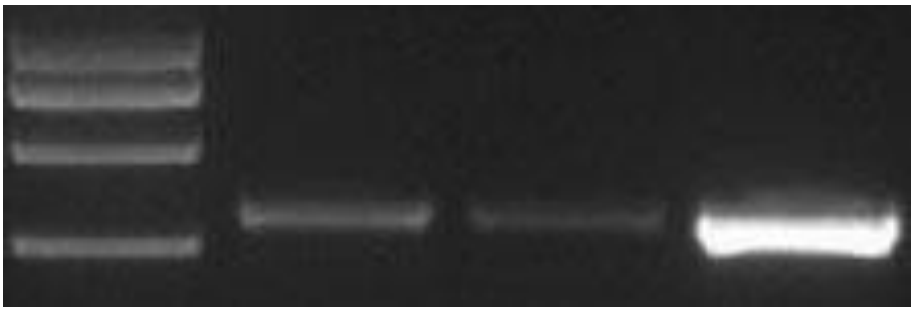

OBP14

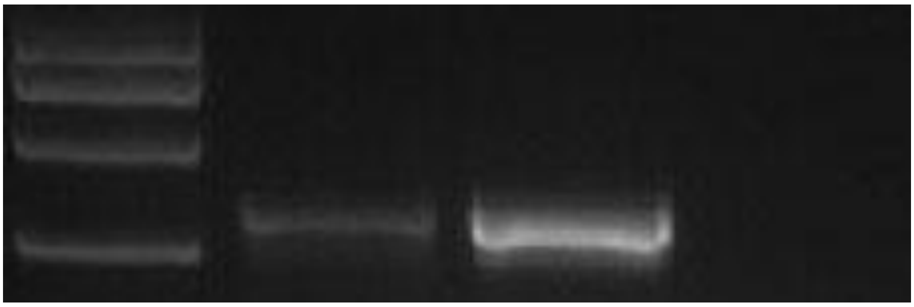

OBP15

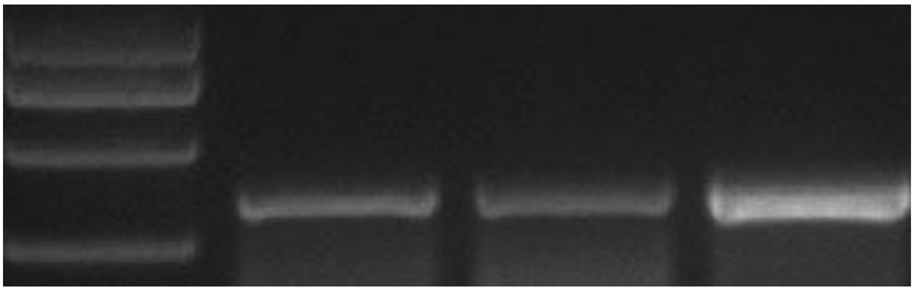

OBP16

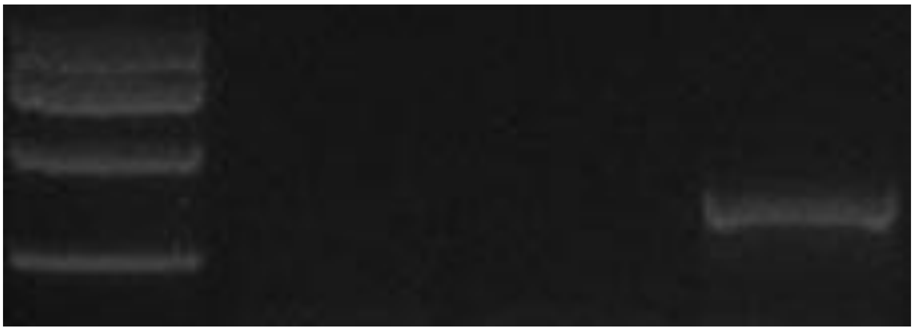

OBP17

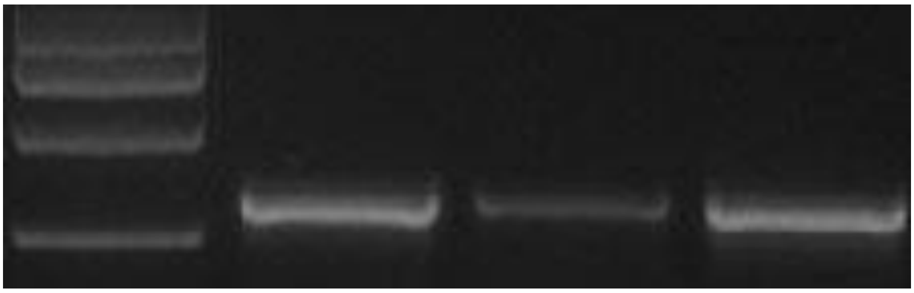

OBP18

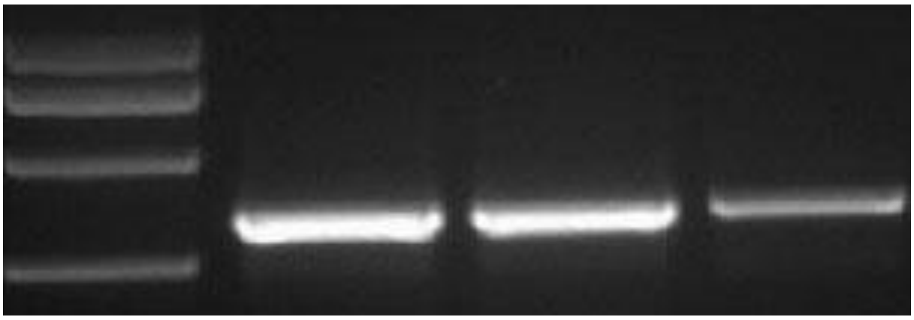

OBP19

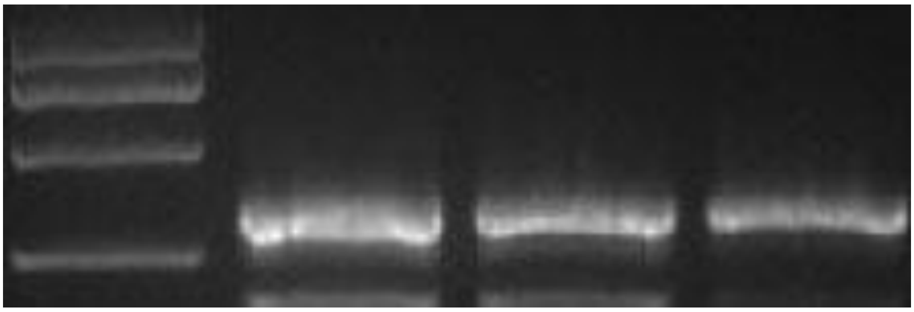

OBP20

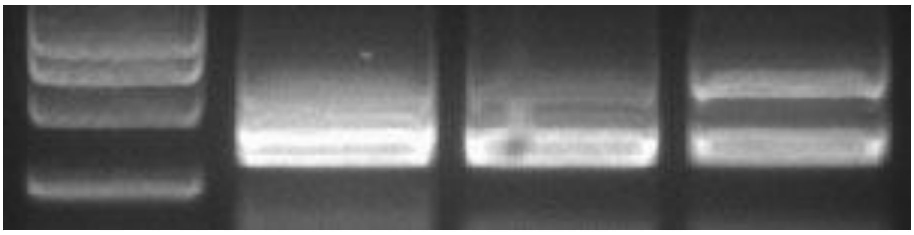

CSP1

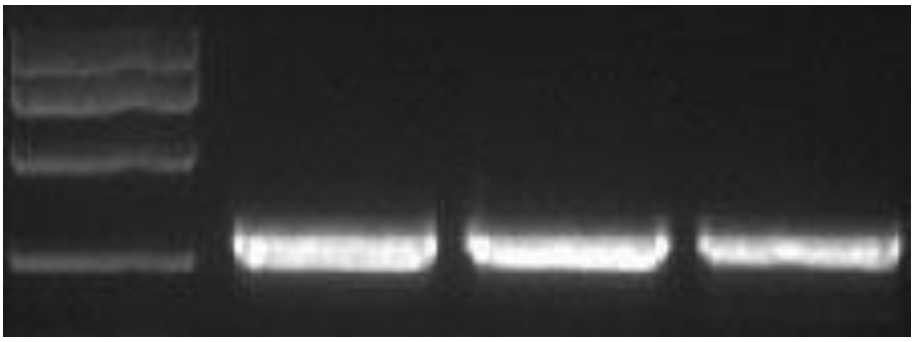

CSP2

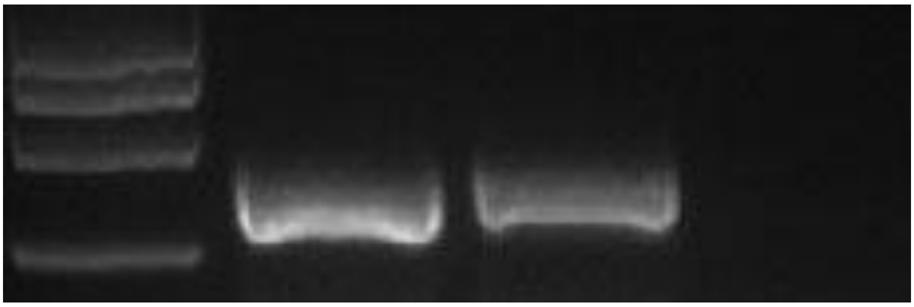

CSP3

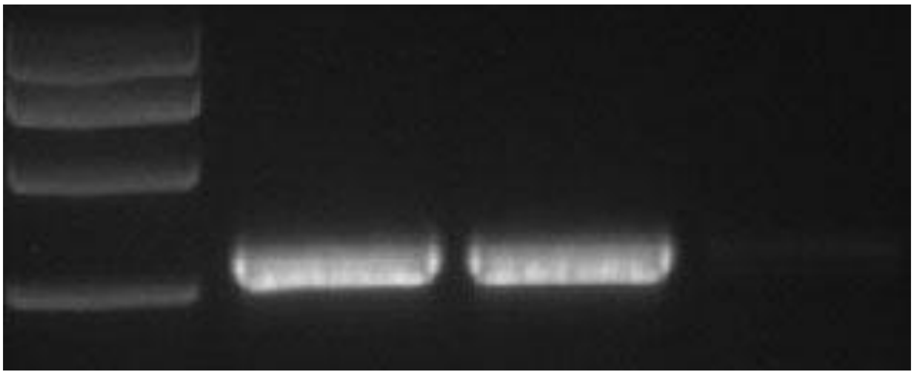

Actin

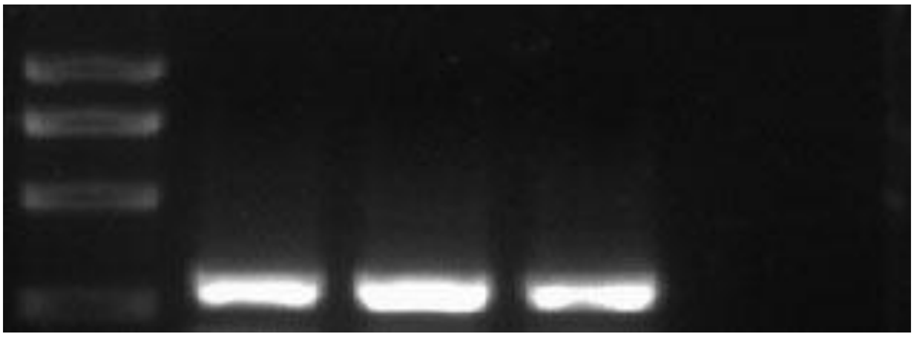

Figure S2.

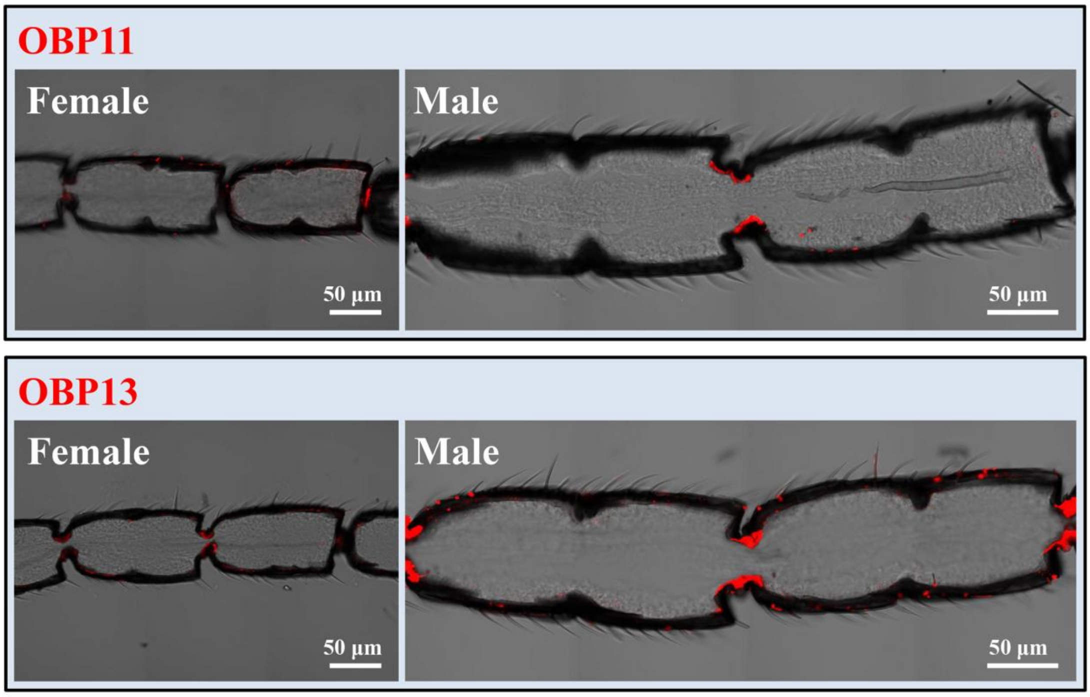

Table S1.

Genes	Forward (5'-3')	Reverse (5'-3')
MmedOBP1	CCACTGGGTAAAACATGTCTAGGA	TGGGAAGAAAACTATCTCTGGACC
MmedOBP2	TCTTAGGAGTTTTGCTGACCGTT	ACTGACAACTCCGTAAACTTGGT
MmedOBP3	GTCAGTTTTAGCGATCGTTGCTT	ACGCCTTATTTTCACGAAGACAC
MmedOBP4	ACCGTAAACAATGCTAGTTCGAA	GCTGCTACGTCACATTCATTCTC
MmedOBP5	CCTAGAAGTTAGAGCACCCTGTT	CTCCGCAGTTTGATTGTAATGCT
MmedOBP6	AATACCCTTTTCTTCACCCTCGC	TTCATGAATTCCCAACTCGGTCT
MmedOBP7	ATTACATTTTCCTCGGCCCTCTT	GATTTGCTCTGGACACATGTAGC
MmedOBP8	CAATCAGACCCACATGCATCAAC	TGGGTCATTTTCAGAGGCTCATT
MmedOBP11	GAGTAGAACTTGACAGACTCGCA	TTTTCAGCTGGGTCTAGTGAGA
MmedOBP12	ATTTGCAACACTACAGATCCCGT	TCGACCGTTACGTCTTGATGATT
MmedOBP13	GCACGGCTTGTATAACTGAAACT	ATAAGCCTCTTTCATCAGCGTGA
MmedOBP14	TTAGTACTGATGCCGCTTTCACT	AAGGATCTTCACTTATCGCTGCA
MmedOBP15	GATGTGATAGGATTTGCTGCGAG	ACGTCTTTGGTGATACTTCTGCA
MmedOBP16	TTCGCTGTATTATTCGCCGTTTG	AACACTTCATCATCAACATGGCG
MmedOBP17	TTCGCTGTAATATTCGCCGTTTG	TTTGCATTTCATCATAACGCCGT
MmedOBP18	TTTCAAGAAAGTATCGACGCGTG	GCTGCGTGTTCCATAAGACATG
MmedOBP19	TCGGTTGTGGTTTTGAGTGAATC	GGGCTGTTTCACATTGGTCATTA
MmedOBP20	TGCTGACATTAAAAGAGATTGCCG	CGGTTGGGCTTTGAAGTAACATT
MmedCSP1	CTTTGGCTGCGACTACTAAAACC	TTACGGTACTCATTATTGGGGTCG
MmedCSP2	CGGAGGCTGTAAATAATAAGGGC	TCATTTCCCTCATTCTGGTCCTG
MmedCSP3	TTTGAATGTTGATGAAGCGCTGG	AATTGGTGATTGACAGATTTGCGT
β-actin	CCATCTACGAGGGTTACGCT	TACCACAGGCTTCCATACCC

Table S2.

>MmedOBP1 (330 bp)
CCACTGGGTAAAACATGTCTAGGAAAAACTGGTCTTAGCAAAGAGGTGCAAGCTGGA
CAACACAATGGCGAATTTCCGGAAGATGAAGCTCTAATGTGTTATCATTCGTGTCTTCTT
AAACTGGCAAAAATCTCTGATAAAAGTGGTAACATTAATTTGGATACTGTACACAAGCA
AATAGATTTAATGATGCCTGAAGATTTAATAGCTCGTGCAAAAGCTGTAACTACAGATT
GTTTTGGTGAAATTAAATCTACGGAAATTTGTAGAATGAGTTTTGAATTTGTCAAATGCT
ATTTCATAAAAGGTCCAGAGATAGTTTTCTTCCCA

>MmedOBP2 (326 bp)
TCTTAGGAGTTTTGCTGACCGTTTTAATTTCTAATAAGGCGGAGGCTAAATCGGTACAG
AAACGAGAATGCCCATTTAAGAAACCATTTGAAGCAAACGCACCCAAATGTATGGACA
AAATATCTGAAGAAAATATGGGCCGTATGATGCAAGGCAATATGGACAATGACGAAATC
CGTTGTTTCGTTGGCTGTGTATTTGAAAACGCCGGTTTTGTGAAAGATAACAAAGTTCA
AATGGACAAAGTTAGAGAGGCCGTTGACAATTTCGTTGACGATTACAAGTATTCAAAA
GAGGTTGGCGACCAAGTTTACGGAGTTGTCAGT

>MmedOBP3 (374 bp)
GTCAGTTTTAGCGATCGTTGCTTGTGCTCTTGTAGTTGGTGTACTGGGAGATGATGACA

TGAAAGAAAAGCACAAGGAAATATTTAAAAAATGTGCGGAAGAAACAGGAGTAACCA
AAGAAGATCTTCATAATCATAAGAGGGGCGAAGAACCCGAGACAAAAATAAAATGTTT
CCATGCTTGTATTGCAAAGGCTGACGGCGCTATGGTCGATGGAAAACTTAATAAAGAC
AAAGTCATCGAGAAGATCCCTGCTGATTTACCAGATCGCGAAAGAATTATTGAAGCCG
TTACAAAATGCAGCGAACAAACGGCAGCTGACGAGTGCGAGACAGCTCATCTTGTTTT
CAAGTGTCTTCGTGAAAATAAGGCGT

>MmedOBP4 (341 bp)

ACCGTAAACAATGCTAGTTCGAATTCAAATATGGAGGAACTTGTCAAAAAATCAATGG
AAGAAACTTTTAAGGCATGCAAAGATAAATTAACCCCAGAAAATTTTGCGCTTCTTAAC
AAGGATCCACACGCTGATAATCAAGAAATTAAATGTTTCAAAGCATGCGGTATGAATCA
TGCGGGTATAATGGCAGACGGTAAAATACAAATTGAAAAAATGGAAGAGAAATTAAAT
TCTTTGTTAGGAGAAGATAAAAAAGACTTTTCAAAAATTATAATTGGACGTGCTAAACC
ATGCGTCGAAGAAGCCAATAAGGGTGAGAATGAATGTGACGTAGCAGC

>MmedOBP5 (331 bp)

CCTAGAAGTTAGAGCACCCTGTTTACATCTGCTATCAAATGAAACACTCACTACATTGA
AAACAAGAAGACACTTAGACAATCCAGAAATTCGGTGTTTCAAAGCATGTTTGATGGA
ACGTCAAGGATATCTTAAAGACAACAAGATCTTCATTGATGAATACGAAAAACTCATTG
ATGTCAATTTGAAGAGAATCAAAGAACTGAACATGAAATTTGCGCGAGCATGTGTTAA
TGAAGCTGAAAAAAGTGAAAATAAGTGTGAGTTAGCACATAATTATAATAGGTGCATTC
TTCATCAGACAAGGAAGCATTACAATCAAACTGCGGAG

>MmedOBP6 (392 bp)

AATACCCTTTTCTTCACCCTCGCTGCGGCTTTTTTATTAGGATATAATATCCCACATGTTG
AATCTAGAATGAGTATGGCACAAACAATAAACACTATGAAGCCATTGGGCAAAACTTG
TGCAGCCAAAACTGGACTTAGTAAAGAGATGCAAGACGGACAACACGAAGGACAATT
TCCAGAAGAGGAAGCTCTTATGTGTTATCACACATGCTTGCTTAAGATGGCTAAAGTTG
CTGACAAAACTGGAAAATTAAATATAGATGCGATGGTTAAACAAATTGATATGTTGATG
CCCGAAGATTTGGTAGACAAAGCAAAAACAGCTTGTTCAGGTTGCGCCGATGAAGTTA
CAGCTACTGAGGGTTGTAGACCGAGTTGGGAATTCATGAA

>MmedOBP7 (385 bp)

ATTACATTTTCCTCGGCCCTCTTTTGCAAACTTTTATCGTTTCTGCGAAGTTACCCGACT
GGGTGCCTGCAGAAATAATTGACATGGCACAAGGAGAGAAGGGTAGATGTATGTCTGA
GCACGGAACAACAGAAGATATGATTAATATGGTTAACGAAGGCAATATTCCTAATGATC
CTAAGTTAACTTGCTACATGTTTTGTCTGTTTGAATCCTTCAGTATAATCGACGAAGACG
GTGTACTGGAATATGGAATGCTGACTGAAATGTTTCCAGATGATATAAAAGCTAAAGCA
GAATCTGTTCTCTCAGGTTGTGCTGAGCAACCCGGAGCTGATAACTGCGAAAAAGTAT
ACAAAATCGCTACATGTGTCCAGAGCAAATC

>MmedOBP8 (377 bp)

CAATCAGACCCACATGCATCAACCCGAAAAAAATGTAGCGGTGAATTTAAATTAACTGA
TGAAATCCTCAAATTAGGAGAACAAGATCCAAGTGATTTTAGCTGTTATCTATTCTGTTT
ATTCAAAGATATTAATATCATGAACCAAAAAGGAGAATTTGATCCTAATCTCGCTGCGC
AAGAAGTACAAGATAATTTGAGGGAAGCCGCTAGGAAATATATATTCATGTGTTATGATT
TAGTCAAACCAAATATGACCAGCGATGGATGCAAAAATGCTCTAGAAATGGTGCAATG
TTTCAAGGAAAAGGCACCTGAAATGTATGAGATGTTAGGACTTTTTCACCCTCCATCAA
ATGAGCCTCTGAAAATGACCCA

>MmedOBP11 (337 bp)
GAGTAGAACTTGACAGACTCGCAAACATTTGTGTAAATGAAACTGGTTTTTACGAGGG
CCACAATTCCGATGATCCGGCTAAAAACTGGATATCGTACGGCTTCAAACTTCAATGTT
ACTTCTCTTGCATGCTGAAGAAAATGAACATCATGAATGAGGACGGAACATTGAACGA
AGAAATGATTCGCAAGAAGATAGGCGACGAAGTGCCAGCAGACAAAATTGACGCCGT
TATTACGAAATGCAAAGACTTGAAGGGCGCTAATAAGTGTGAGACCGCCACCATGATA
ATGAAGTGTTACAGTGATGAGAGACTCTCACTAGACCCAGCTGAAAA
>MmedOBP12 (323 bp)
ATTTGCAACACTACAGATCCCGTTGATTTACGAGTATTGAATGATTATTTGATGAATCAT
AATTTAAATCGCCTGCACATAAAATCACACCACCCACTTGCCTGTTTCCTACTCTGTGT
GTACAGTGAATTCAATTGGATGGATCGTCATGGGGGATTCAAAGTTCATAATATCAAGG
CCTGGATGCTAAGAGCTGAATTATCGGAAAATGACACGGATATTTTATTGAGAAAGTGC
ATTAGCTTAGAGTTGACAGATCCTTGTACACGGGCGCAATATTTTACTGAATGTTTTTGG
ACAAATCATCAAGACGTAACGGTCGA
>MmedOBP13 (303 bp)
GCACGGCTTGTATAACTGAAACTCTTGTTGATGACGCCAACGTTGACGCAGCTATGCAT
CACAATATCTGGAGAATGGACGACCTTAAACTTCGGTGTTATTTCTTTTGCCTGCTGAA
AAAACTCAAAGTCATGAATGAGGATGGAAAATTGAACGAGGAGATCACTCGCCAGAG
GTTGGCCAACCTTTTCCCAGCAGACAGAATTGACGGCGTTATCATGAAATGCAAAGAA
ATGAAGGGCGCTGATGCCTGCGAGACCGCAATATTGATGGCTAAGTGTCACGCTGATG
AAAGAGGCTTAT
>MmedOBP14 (315 bp)
TTAGTACTGATGCCGCTTTCACTGTAGAACAAATAGAGAGTATGATGAAACCACTGGGT
AACAATTGCGTGTCAAAAGTTGGGCTCAGTCCAGAGTTACAAGAGGCAAATAGAAAA
AAAGAATTTCCAGAAGAAAAACCCTTCATGTGTTATCTTCATTGTCTTGCAAGAGTGAC
GAAAGTTTTTGACAAAAATAATCAAATTGATTTAGAGGGTACATTAAAACAAGTAAGAT
TAGTAATGCCAGACCATTTAGTAGAGGGATCAGTAAAAGCCTACACAGTTTGTTCTCGT
GCAGCGATAAGTGAAGATCCTT
>MmedOBP15 (343 bp)
GATGTGATAGGATTTGCTGCGAGTGGAGTTAATGCATGTCAAAGACAAACAGGCGTGG
CTACTGCGGACATTGAAGCTGTACGGAATGGACAGTGGCCAGAGTCTCGCCAATTAAA
ATGTTACATGTACTGTCTTTGGGAACAATTTGGATTAATTGATGAAAAAGGCGAATTAA
GTCTAAACGGAATGTTAACATTTTTTCAAAGAATACCAGCCTACAGAGTTGAAGTACAA
AAAGCTATTCGCGAATGCAAGAGTATCGGTGAATATTTAGCGAACGGAGATAATTGTCA
GTACGCATTTACATTTAATATGTGTTATGCAGAAGTATCACCAAAGACGT
>MmedOBP16 (361 bp)
TTCGCTGTATTATTCGCCGTTTGCTTCGTTGGTGCTCTAGCAGAGCTCACTCCGGAACA
ATTGGCTAAGCTCCACGAATCCAGATCAACTTGTATAACTGAAACTGGTGTTGAAGAA
GGAAACGTTGCCAAGGCCAATGATGGCGAATGGTTGATGGACGACCTCAAACTTCGGT
GTTTCTTCTCTTGCATGCTGAAGAAAATAAAAGTCCTGAATGAGGATGGAACTTTCAAC
GAAGAGAAAGCTCGCAAGCGGATAGCCAACGATCTGCCAGCAGACAAAATCGATTCT
GTTATCACGAAATGCAAAGACTTGAGCGGCGGTGATGTTTGTGAGACCGCCATGTTGA
TGATGAAGTGTT
>MmedOBP17 (309 bp)

TTCGCTGTAATATTCGCCGTTTGCTTCGTTGCTGCTCTGGCCGAGCTCACTGAAGAACA
AAAAGCTAAACTCCGCGAACACAGAACCGCTTGTGTAACTGAAACTGGTGTCGATGA
AGCCAACGTTGACGCAGCCAAGCAAGGCGATTGGAAGATGGACGACCTTAAGCTTCG
GTGTTTCTTCGCTTGCATGATGAAGAAAATAAAAGTCATGAATGAGGATGGAACACTG
AACGAAGAGATAACTCGCAAGAGGATGGCCAACGATCTGCCAGCAGACAAAATCGAC
GGCGTTATGATGAAATGCAAA

>MmedOBP18 (338 bp)

TTTCAAGAAAGTATCGACGCGTGCAAGGATAAACTATCAGAAGAAAACCTGGCTCTCC
TGGAGAAAGATGAGAACGCTGACAATGAGGATATCCGATGTTTCAAGGCATGCATTTT
GAACGACAGTGGTGTGATGAGTAATGGTAAAATCCAGATTGATAAAATTGAAGAAGCA
ATCAATGCTGCTATTGAAAATGTAAAAGAAGACGAAGAGAAAGCTAAAGCAATCGGA
GAATCTATGATTAACGGTGCTAAAAATTGCGCTGGACCAGCTGAAGAAGGTGAAAATG
AATGTGAAGTAGCACACCGTTTTATTACATGTCTTATGGAACACGCAGC

>MmedOBP19 (325 bp)

TCGGTTGTGGTTTTGAGTGAATCGGCGATAACTGCCGAAGATCTTGTGAAATTTGGAAT
GGCTCGTAGAACATGCGACCGTACCAATAGAGTCGATCCCAGTGTAATCGATCGTGTAC
TCCAAGGCGAAATGATAAACGACCCCCAGTTTGATTGCCACGTCGCTTGTGTTTTGAA
GGAATTAAATTTGTTGACTGCAGACGGTTCTCTGAATGTTGAAGTAGCCGCCAGTAAA
GTTCCTGAAAATTTACCATACTACAATCAGCTTGTCGGTGCAATCAGATCATGTGGCAG
CAGAAAGGGTAATGACCAATGTGAAACAGCCC

>MmedOBP20 (301 bp)

TGCTGACATTAAAAGAGATTGCCGTAAACAAACTGGCGTATCTTGGGCTTCATTGAAA
AAACTAAAGGCGGCAGATTATAATCAGAATGATCCTAAATTGAAGTGCTACTTGAAATG
TTTCATGCAAAAAAACGGAATATTTGGAGAAGACGATATCGATATTGAAAAAGCACTG
AGGCATTTACCGACAGGCATTAAAGGCCCTTCGAAAACGACACTAGAATATTGCAAAA
AAATTCCTAGTGTAGATTCATGTGACAAAGCATTTCAACTCGCAAAATGTTACTTCAAA
GCCCAACCG

>MmedCSP1 (301 bp)

CTTTGGCTGCGACTACTAAAACCTACACCAGTAAATTCGATGATGTTGACGTTGATGGT
ATTTTAGGCAGTGATCGCTTATTAAGAAATTACGTTAATTGTTTGTTAGACAGAGGACCT
TGTACTAAAGAGGGAGTCACATTAAAAGAGATTTTACCTGATGCTCTTGCCACTAGTTG
TGAAAGCTGCACTGAAAAGCAAAAGACTAAAAGTGAAAAAGTTATCCGACACTTGGT
CAATAACAAAAAAGAATTGTGGGATGAATTAGCTGTCAAATACGACCCCAATAATGAGT
ACCGTAA

>MmedCSP2 (352 bp)

CGGAGGCTGTAAATAATAAGGGCATGTACTCAACAAAGTATGACAATATTGATATCAAC
GCGATTATCAAAAATGAACGGCTACTCAATAATTATGTCGGCTGTCTAATGGACGAGAA
ACCCTGCACTCCAGATGGTGCTGAGCTTAAAAAAAATCTTCCAGATGCTCTGGCAAGT
GAGTGCGCAAGTTGCAGTCCAGCACAAAAAAATATCGCTAATGTGATGTATCATCATCT
GATTGATAATCGTCCGGATTTGTGGTCTAAGTTGGAGACTAAATACGACCCATCAGGTG
GTTACAGAAAACGTTATCTCAACCAGGACCATGACCAGGACCAGAATGAGGGAAATG
A

>MmedCSP3 (301 bp)

TTTGAATGTTGATGAAGCGCTGGCTAATGCCGAAGTAAGACAGACTTATTTAATTGTT

TTATGGATAAAGGGCCATGTGGAGAAGACGCGACTTATTGGAAAGGTAATTTTCCCGA
AGCGATAGCGACAAATTGCAAAAAATGCACCGAATGGCAGAAAGAAGCATTTGACAA
AATCGCCGACTGGTACACAGTCCATGAGCCAGACAACTGGAATTCGTTCGTCGACAAA
ATGGTACAAGGAGCCCGAAACTTCGGCGATAGCAGGAAATGATTAACGCAAATCTGTC
AATCACCAATT
